# Unified understanding of folding and binding mechanisms of globular and intrinsically disordered proteins

**DOI:** 10.1007/s12551-017-0346-7

**Published:** 2018-01-06

**Authors:** Munehito Arai

**Affiliations:** Department of Life Sciences, Graduate School of Arts and Sciences, The University of Tokyo, 3-8-1 Komaba, Meguro, Tokyo, 153-8902 Japan

**Keywords:** Protein folding, Intrinsically disordered protein, Ligand binding, Conformational selection, Induced-fit, Nucleation–condensation

## Abstract

Extensive experimental and theoretical studies have advanced our understanding of the mechanisms of folding and binding of globular proteins, and coupled folding and binding of intrinsically disordered proteins (IDPs). The forces responsible for conformational changes and binding are common in both proteins; however, these mechanisms have been separately discussed. Here, we attempt to integrate the mechanisms of coupled folding and binding of IDPs, folding of small and multi-subdomain proteins, folding of multimeric proteins, and ligand binding of globular proteins in terms of conformational selection and induced-fit mechanisms as well as the nucleation–condensation mechanism that is intermediate between them. Accumulating evidence has shown that both the rate of conformational change and apparent rate of binding between interacting elements can determine reaction mechanisms. Coupled folding and binding of IDPs occurs mainly by induced-fit because of the slow folding in the free form, while ligand binding of globular proteins occurs mainly by conformational selection because of rapid conformational change. Protein folding can be regarded as the binding of intramolecular segments accompanied by secondary structure formation. Multi-subdomain proteins fold mainly by the induced-fit (hydrophobic collapse) mechanism, as the connection of interacting segments enhances the binding (compaction) rate. Fewer hydrophobic residues in small proteins reduce the intramolecular binding rate, resulting in the nucleation–condensation mechanism. Thus, the folding and binding of globular proteins and IDPs obey the same general principle, suggesting that the coarse-grained, statistical mechanical model of protein folding is promising for a unified theoretical description of all mechanisms.

## Introduction

Elucidation of the mechanisms of protein folding and function remains an outstanding challenge in biophysics. Extensive experimental and theoretical studies have greatly advanced our understanding of the folding mechanisms of globular proteins (Dill and Chan 1997; Arai and Kuwajima 2000; Daggett and Fersht 2003; Englander and Mayne 2014; Takahashi et al. 2016), although many fundamental problems remain unsolved (Dill et al. 2008; Sosnick and Barrick 2011). Recent studies have also revealed that proteins disordered in isolation fold into specific structures upon binding to their partners. Because more than 30% of eukaryotic proteins have disordered regions of over 30 residues in length and participate in critical cellular control mechanisms, including transcription, translation, and cell cycle control, these proteins are categorized as intrinsically disordered proteins (IDPs) (Wright and Dyson 1999; Dunker et al. 2001). The mechanisms of coupled folding and binding of IDPs have been extensively studied (Dyson and Wright 2005; Wright and Dyson 2009, 2015; Tompa 2012; Mollica et al. 2016). In addition, recent advances in nuclear magnetic resonance (NMR) and fluorescence techniques have provided a detailed understanding of the dynamic motions of globular proteins during ligand binding and catalysis over multiple time scales, ranging from picoseconds to seconds or longer (Mittermaier and Kay 2006; Henzler-Wildman and Kern 2007; Banerjee and Deniz 2013). However, the folding and binding mechanisms of globular proteins and IDPs are typically discussed separately, and few studies have attempted to integrate these mechanisms (Kumar et al. 2000; Tsai et al. 2001; Liu et al. 2012; Chen et al. 2015). Although folding reactions of globular proteins and IDPs are induced by intramolecular and intermolecular interactions respectively, the forces responsible for conformational changes and binding are common to both proteins. Therefore, it may be possible to comprehensively understand the mechanisms of coupled folding and binding of IDPs, folding of small and multi-subdomain proteins, folding of multimeric proteins, and ligand binding of globular proteins.

In this review, we attempt to integrate the folding and binding mechanisms of globular proteins and IDPs in terms of conformational selection and induced-fit mechanisms (Boehr et al. 2009; Csermely et al. 2010). The two mechanisms have been widely used to understand the mechanisms of coupled folding and binding of IDPs and ligand binding of globular proteins, but have not been used to describe the folding mechanisms of globular proteins. We also reinterpret and synthesize folding mechanisms of small and multi-subdomain proteins, regarding the protein-folding reaction as the binding reaction of intramolecular segments accompanied by secondary structure formation. In the following, we describe conformational selection and induced-fit mechanisms and discuss mechanisms of coupled folding and binding of IDPs, folding of monomeric globular proteins, folding of multimeric proteins, and ligand binding of globular proteins. Finally, we aim to obtain a unified understanding of these mechanisms.

## Conformational selection and induced-fit mechanisms

**Fig. 1:**
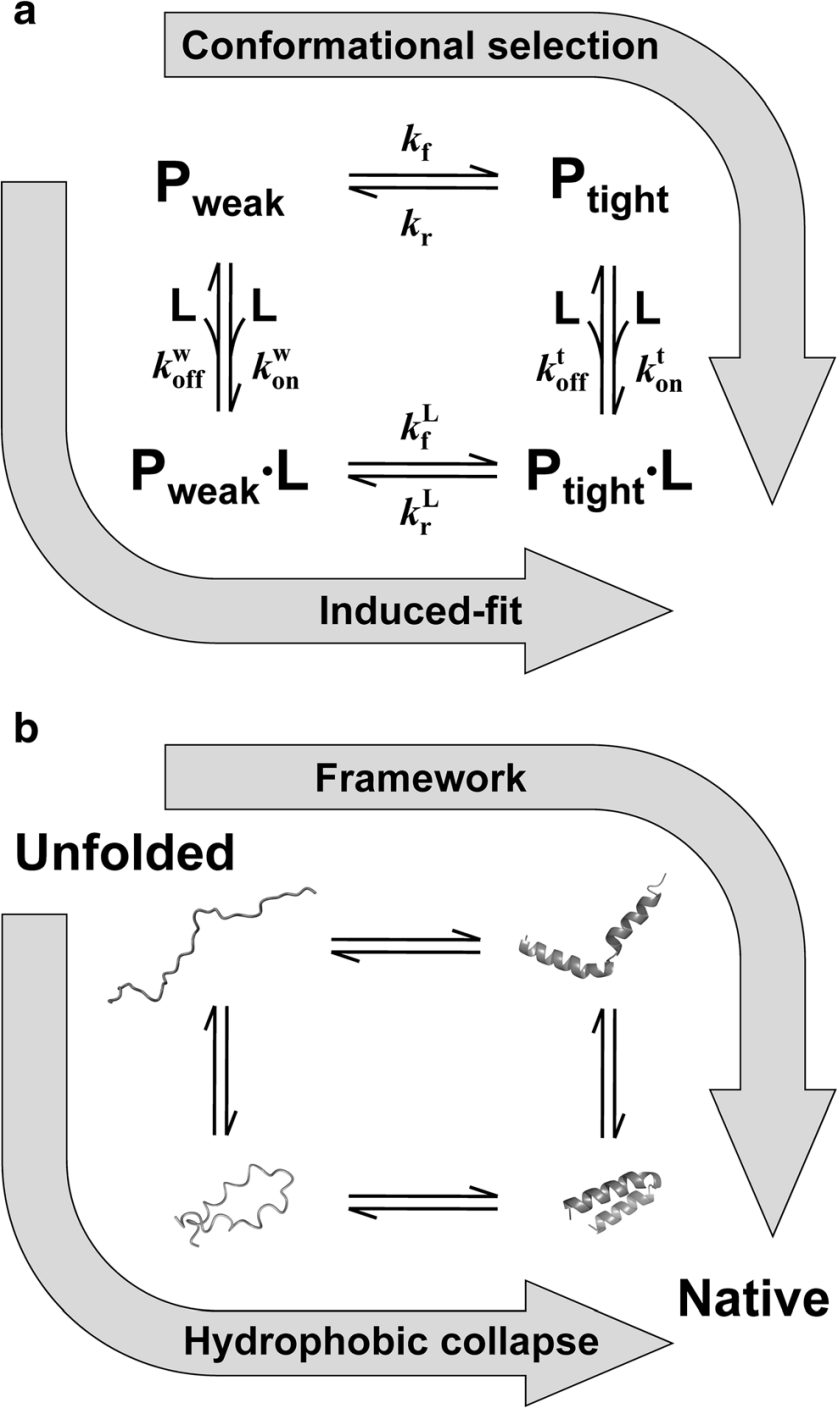
**a** Conformational selection and induced-fit mechanisms. P_weak_ and P_tight_ denote weakly and tightly binding conformations respectively, and L denotes a ligand. *k*_on_ and *k*_off_ denote the second-order binding rate constant and first-order dissociation rate constant respectively. *k*_f_ and *k*_r_ denote the forward and reverse rate constants respectively. **b** Two representative mechanisms of protein folding. The framework model corresponds to the conformational selection mechanism, while the hydrophobic collapse model corresponds to the induced-fit mechanism

The conformational selection and induced-fit mechanisms are two common mechanisms that explain binding reactions accompanied by conformational changes of proteins (Fig. 1a). Recent studies have demonstrated the existence of dynamic motions of proteins both in ligand-free and ligand-bound forms, underlying the dynamic binding mechanisms rather than the lock-and-key mechanism. The mechanism in which conformational change precedes binding is known as the *conformational selection* mechanism (or population-shift mechanism, pre-existing mechanism, folding-before-binding mechanism), while the mechanism in which binding precedes conformational change is known as the *induced-fit* mechanism (or binding-before-folding mechanism) (Monod et al. 1965; Koshland et al. 1966; Ma et al. 1999; James and Tawfik 2003; Onitsuka et al. 2008; Boehr et al. 2009; Csermely et al. 2010; Changeux 2012; Vogt et al. 2014). The induced-fit mechanism assumes that after weak binding to a ligand, a protein undergoes conformational change from the weakly bound conformation (P_weak_·L) to the tightly bound conformation (P_tight_·L) to fit the ligand. Recent studies have shown that many IDPs bind their partners through the induced-fit mechanism (Wright and Dyson 2009; Mollica et al. 2016). In contrast, the conformational selection mechanism assumes that equilibrium between the weakly binding conformation (P_weak_) (or binding-incompetent conformation) and tightly binding conformation (P_tight_) pre-exists, and that a ligand selectively binds P_tight_. Recent advances in experimental studies using NMR spectroscopy and theoretical studies using molecular dynamics simulations have revealed that many globular proteins have low-populated, excited states that can bind ligands (Henzler-Wildman and Kern 2007; Boehr et al. 2009). In this mechanism, the population of P_tight_ is not necessarily lower than that of P_weak_ if conformational equilibrium exists.

At one extreme, the *“ideal” induced-fit* mechanism assumes that a ligand must bind P_weak_ before the formation of P_tight_∙L. Examples of this mechanism are a protein that cannot fold without a ligand, a protein with an extremely fast binding rate, and a protein that sequesters a ligand-binding site in P_tight_, as a ligand cannot bind an inaccessible site buried deep inside of a protein. At the other extreme, the *“ideal” conformational selection* mechanism assumes that conformational changes of a protein must precede ligand binding, and that equilibrium pre-exists between the binding-incompetent and binding-competent conformations. Examples of this mechanism are a protein that cannot form a ligand-binding site before conformational change, a protein that cannot fold after binding, and a protein that has an extremely fast rate of conformational change, exceeding the apparent binding rate of the diffusion-controlled limit, even at high ligand concentrations.

Between these two extremes, the observed reaction mechanism is determined by competition of the fluxes of the conformational selection and induced-fit pathways (Hammes et al. 2009; Daniels et al. 2014; Greives and Zhou 2014) (Fig. 1). A flux is determined by both the rate constants and concentrations of all species involved in a reaction pathway. Thus, simple comparison of the rate of conformational change and second-order binding rate constant may not accurately reveal which pathway is dominant. Instead, the rate of conformational change and apparent binding rate of interacting elements determine the reaction mechanisms (Hammes et al. 2009; Greives and Zhou 2014). The former is affected by both the forward rate (P_weak_ to P_tight_) and reverse rate (P_tight_ to P_weak_), while the latter is affected by the second-order binding rate constant, first-order dissociation rate constant, and protein and ligand concentrations. The flux description suggests that if protein and ligand concentrations are low or if the conformational change from P_weak_ to P_tight_ is fast, the flux of the induced-fit pathway is small. Consequently, most protein molecules go through the conformational selection pathway, while a small fraction of protein molecules can go through the induced-fit pathway. Thus, the observed mechanism should be referred to as the *“apparent” conformational selection* mechanism. In contrast, if protein and ligand concentrations are high or if the conformational change from P_weak_ to P_tight_ is slow, the flux of the induced-fit pathway is large. Consequently, most protein molecules go through the induced-fit pathway, while a small fraction of protein molecules can go through the conformational selection pathway. Thus, the observed mechanism should be referred to as the *“apparent” induced-fit* mechanism. Therefore, the conformational selection and induced-fit mechanisms can coexist. Because few examples of the “ideal” mechanisms have been described, the reaction mechanisms correspond to the “apparent” mechanisms in many cases and depend on protein and ligand concentrations. This indicates that assignment of a single mechanism to a single protein is impossible in many cases. However, if experiments are carried out under similar conditions for different proteins, comparison of the “apparent” reaction mechanisms can reveal the intrinsic characters of the proteins.

A more complicated reaction mechanism, known as the *extended conformational selection* mechanism (Csermely et al. 2010), involves initial ligand binding by the conformational selection mechanism followed by subsequent conformational change by the induced-fit mechanism (James and Tawfik 2003, 2005; Tang et al. 2007; Boehr et al. 2009; Espinoza-Fonseca 2009; Wlodarski and Zagrovic 2009; Wang et al. 2013a; Schneider et al. 2015). Furthermore, different regions of a single protein can adopt different reaction mechanisms (Arai et al. 2015).

## Mechanisms of coupled folding and binding of IDPs

Coupled folding and binding reactions of IDPs have been studied experimentally using various methods, including NMR relaxation dispersion measurements which reveal dynamic conformational changes on microsecond to millisecond time scales with a residue-specific-level spatial resolution (Mittermaier and Kay 2006; Gibbs and Showalter 2015). Additionally, many theoretical studies have been performed to explain experimental results and provide insight into the reaction mechanisms (Chen et al. 2015). The results revealed that many IDPs bind their partners by the induced-fit mechanism (Wright and Dyson 2009; Shammas et al. 2016; Mollica et al. 2016). The best studied example is the kinase-inducible domain (KID) of cAMP response element binding (CREB) protein, which binds the KIX domain of a CREB-binding protein (CBP) (Dyson and Wright 2005, 2016; Wright and Dyson 2015). Post-translational modifications of IDPs, such as phosphorylation, can modulate interactions with their partners by changing electrostatic interactions and/or by inducing the folding of IDPs (Forman-Kay and Mittag 2013; Bah and Forman-Kay 2016). Phosphorylation of KID (pKID) slightly stabilizes its free form, but largely increases its affinity for KIX by enhancing electrostatic attractions (Radhakrishnan et al. 1998). NMR relaxation dispersion experiments showed that intrinsically disordered pKID binds KIX by the induced-fit mechanism with accumulation of an intermediate (Sugase et al. 2007). Coupled folding and binding reactions by the induced-fit mechanism have also been reported for many other IDPs, including the transactivation domains (TADs) of c-Myc, Gal4, and VP16 (Ferreira et al. 2005), CBD of WASP binding to Cdc42 (Lu et al. 2007), IA_3_ binding with YPrA (Narayanan et al. 2008), S-peptide binding with S-protein (Kiefhaber et al. 2012), N_TAIL_ domain from Sendai virus nucleoprotein binding to phosphoprotein PX (Wang et al. 2013b; Dosnon et al. 2015; Schneider et al. 2015), PUMA binding with MCL-1 (Rogers et al. 2014), BimBH3 binding with BAX (Jhong et al. 2016), and STAT2 TAD binding with the TAZ1 domain of CBP (Lindstrom and Dogan 2017).

**Fig. 2:**
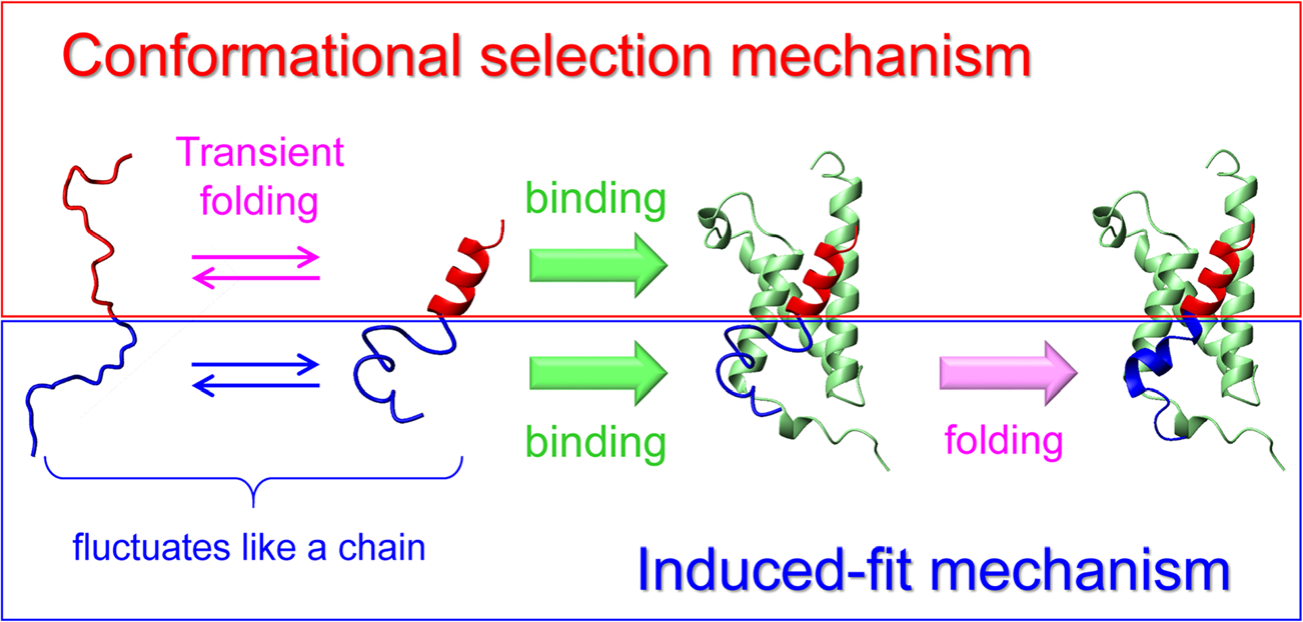
Mechanisms of the coupled folding and binding reaction of intrinsically disordered c-Myb TAD upon binding to KIX (*green*). The N-terminal region of c-Myb TAD (*red*) binds KIX by the conformational selection mechanism (*upper*), while the C-terminal region of c-Myb TAD (*blue*) interacts with KIX by the induced-fit mechanism (*lower*)

In contrast, few studies have reported IDPs that bind partners by the conformational selection mechanism (Onitsuka et al. 2008; Song et al. 2008; Iešmantavičius et al. 2014; Schneider et al. 2015). However, a combination of NMR and mutational analyses showed that the TAD of c-Myb binds the KIX domain of CBP mainly via the conformational selection mechanism (Giri et al. 2013; Arai et al. 2015). Mutation data were interpreted based on the following assumptions. In the case of conformational selection, mutations that stabilize the tightly binding conformation of an IDP should increase its population and accelerate the overall reaction rate. In contrast, in the case of the induced-fit mechanism, mutations that destabilize the tightly binding conformation of an IDP should increase the population of the weakly binding conformation, which can bind a partner, and accelerate the overall reaction rate. When mutations were introduced to stabilize the helical structure in the N-terminal region of c-Myb TAD, the overall reaction rate linearly increased with predicted helix stability (Arai et al. 2015). These results indicate that the N-terminal region of c-Myb TAD binds KIX through the conformational selection mechanism. Furthermore, mutations in the C-terminal region of c-Myb TAD that destabilize the helical structure increased the overall reaction rate, indicating that the C-terminal region interacts with KIX by the induced-fit mechanism (Arai et al. 2015). Thus, c-Myb provides an interesting example of an IDP in which two different reaction mechanisms coexist in a single protein (Fig. 2).

The above results show that pKID and c-Myb bind the same site on the same target protein KIX, but with different reaction mechanisms. Although both have similar second-order binding rate constants of 10^6^–10^7^ M^−1^ s^−1^, their rates of folding (conformational change) were different; it takes ~1 ms for pKID folding in the KIX-bound form and < 60 μs for c-Myb folding in the free form (Sugase et al. 2007; Arai et al. 2015). Therefore, the difference in the reaction mechanism is probably related to differences in the folding rate, and a faster (or slower) folding rate results in the conformational selection (or induced-fit) mechanism. This is consistent with theoretical studies, which suggested that the rate of conformational change can determine the reaction mechanisms (Greives and Zhou 2014). Prediction of helical propensity indicated that c-Myb and pKID have high and low helical propensities (Arai et al. 2015), which are consistent with the fast and slow folding rates respectively. Such conformational propensities may depend on protein function. c-Myb is a constitutive transcriptional activator expected to bind its partner as soon as it is synthesized, and thus, it has high helical propensity to fold and bind quickly. In contrast, pKID is an inducible transcriptional activator that tightly binds KIX only after it is phosphorylated. The high degree of disorder and low propensity for secondary structure formation of pKID may facilitate interactions with and phosphorylation by protein kinase A, which binds peptide substrates in a relatively extended conformation (Zor et al. 2002). Therefore, function determines conformational propensity, conformational propensity determines folding rate, and folding rate determines the reaction mechanism of c-Myb and pKID (Arai et al. 2015).

Laser-induced temperature jump experiments showed that α-helices and β-hairpins are formed with a time constant of 0.2–2 and 0.1–6 μs respectively (Kubelka et al. 2004; Muñoz and Cerminara 2016). However, because these measurements were conducted using stable secondary structure elements, the folding of intrinsically disordered regions (IDRs) will occur over longer time scales, as indicated by the marginal stability of isolated secondary structure elements (Sadqi et al. 2003; Sugase et al. 2007; Arai et al. 2015). Moreover, some IDPs form single β-strand or irregular structures upon binding to their partners, but such structures cannot be stabilized without partners. Thus, few IDPs have stable secondary structure elements that can fold rapidly. Consequently, coupled folding and binding reactions of IDPs are dominated by the induced-fit mechanism.

Second-order binding rate constants of IDPs have been reported to be 10^5^–10^10^ M^−1^ s^−1^ (Sugase et al. 2007; Arai et al. 2012, 2015; Zhou and Bates 2013; Dogan et al. 2014; Milles et al. 2015; Shammas et al. 2016). We recently found that the intrinsically disordered N-terminal activation domain 2 of tumor suppressor p53 interacts with the TAZ2 domain of CBP with an extremely fast binding rate of 1.7 × 10^10^ M^−1^ s^−1^ (Ferreon et al. 2009; Lee et al. 2010; Arai et al. 2012). This is the fastest rate among all previously known protein–protein associations. Although extended structures of IDPs were predicted to enhance binding rates by the “fly-casting mechanism” (Shoemaker et al. 2000), the large capture radius of IDPs also leads to slower translational diffusion, which opposes rapid binding (Huang and Liu 2009). Instead, favorable electrostatic attractions can dramatically accelerate binding reactions (Berg and von Hippel 1985; Schreiber 2002). p53 activation domain 2 and TAZ2 are negatively (−8) and positively (+14.3) charged respectively, and interact with each other through strong electrostatic attractions, leading to a binding rate close to the diffusion-controlled limit (Berg and von Hippel 1985; Arai et al. 2012). Consistently, theoretical studies showed that long-range electrostatic interactions are necessary for the rapid association and formation of initial encounter complexes (Ganguly et al. 2012; Wong et al. 2013; Chu et al. 2017; Ou et al. 2017). Although IDPs tend to be deficient in hydrophobic residues, the regions that directly interact with their partners often contain hydrophobic residues (Meszaros et al. 2007; Arai et al. 2010, 2012; Forman-Kay and Mittag 2013). Therefore, whereas long-range electrostatic interactions are important for attracting an IDP close to its partner, hydrophobic interactions stabilize direct contacts between them (Arai et al. 2012; Ganguly et al. 2012; Wong et al. 2013). These results also suggest that although hydrophobic interactions are reported to be long-range (Meyer et al. 2006), electrostatic interactions are more effective at longer ranges than are hydrophobic interactions.

## Folding mechanisms of globular proteins

### Multi-subdomain proteins

Experimental studies on protein folding have shown that folding behaviors differ between small single-domain proteins of less than 100 residues and multi-subdomain proteins of more than 100 residues (Jackson 1998; Arai and Kuwajima 2000; Daggett and Fersht 2003; Englander and Mayne 2014; Takahashi et al. 2016). Kinetic folding mechanisms of multi-subdomain proteins have been well studied for α-lactalbumin (α-LA) (Kuwajima 1989; Arai and Kuwajima 1996; Chaudhuri et al. 2000; Yoda et al. 2001; Arai et al. 2002; Saeki et al. 2004), non-Ca^2+^-binding lysozymes (hen and human lysozymes) (Matagne and Dobson 1998; Arai et al. 2000), Ca^2+^-binding lysozymes (equine and canine lysozymes) (Mizuguchi et al. 1998; Nakamura et al. 2010), apomyoglobin (Dyson and Wright 2017; Nishimura 2017), barnase (Fersht 1993), dihydrofolate reductase (DHFR) (Jennings et al. 1993; Arai et al. 2003b, c, 2007, 2011; Arai and Iwakura 2005), β-lactoglobulin (Kuwajima et al. 1996; Arai et al. 1998; Fujiwara et al. 1999; Forge et al. 2000; Kuwata et al. 2001), cytochrome *c* (Takahashi et al. 1997; Akiyama et al. 2000; Winkler 2004; Goldbeck et al. 2009; Kathuira et al. 2014; Hu et al. 2016), ribonuclease A (Kim and Baldwin 1982; Neira and Rico 1997; Wedemeyer et al. 2000; Kimura et al. 2005), ribonuclease H (Raschke and Marqusee 1997; Hu et al. 2013; Rosen et al. 2014), and tryptophan synthase α-subunit (Wu et al. 2008). These proteins accumulate a kinetic intermediate(s), resembling a molten globule state, during the folding reaction from the unfolded state to the native state (Kuwajima 1989; Kim and Baldwin 1990; Matthews 1993; Ptitsyn 1995; Arai and Kuwajima 2000; Bilsel and Matthews 2000, 2006; Baldwin 2008; Englander and Mayne 2014; Takahashi et al. 2016). Thus, the folding reaction of multi-subdomain proteins consists of at least three states and two steps. The molten globule state has a compact, globular structure (“globule”) with a pronounced secondary structure, but has little, if any, tertiary structure, as exemplified by the presence of only a small amount of tight packing of side chains (“molten”) (Kuwajima 1989; Ptitsyn 1995; Arai and Kuwajima 2000). For some proteins, including α-LA, Ca^2+^-binding lysozymes, apomyoglobin, cytochrome *c*, and ribonuclease H, the molten globule states are observed under equilibrium conditions, such as at low pH, moderate concentrations of denaturants, and moderate temperatures, and have been shown to be equivalent to the kinetic folding intermediates (Kuwajima 1989; Ptitsyn 1995; Raschke and Marqusee 1997; Mizuguchi et al. 1998; Arai and Kuwajima 2000; Nakao et al. 2005). Because the hydrophobic core present in the molten globule state is “wet” and exposed to solvent, water-separated hydrophobic interactions can exist, in addition to direct contacts between hydrophobic residues (Pratt and Chandler 1986; Arai and Kuwajima 1996). Protein folding from the molten globule to the native state occurs by the exclusion of water molecules through the “dry” molten globule state (Baldwin et al. 2010).

Two representative models have been postulated as the folding mechanisms of globular proteins (Arai and Kuwajima 1996): the *framework* model (or secondary structure coalescence model) (Kim and Baldwin 1982, 1990) and *hydrophobic collapse* model (Dill 1990; Dill et al. 1995). In the framework model, the formation of a secondary structure framework precedes compaction of a protein molecule through interactions between secondary structure elements, followed by formation of tight packing of side chains. In contrast, in the hydrophobic collapse model, compaction of a protein molecule by hydrophobic interactions precedes the formation of secondary structure elements and tight packing of side chains. The driving force of protein folding is the local secondary structure propensities in the former and non-local hydrophobic interactions in the latter. In terms of conformational selection and induced-fit mechanisms (Tompa 2012; Chen et al. 2015), the framework model corresponds to a conformational selection mechanism, as secondary structure formation precedes binding of intramolecular segments (Fig. 1b). In contrast, the hydrophobic collapse model corresponds to an induced-fit mechanism, as compaction by binding of intramolecular segments precedes secondary and tertiary structure formation (Fig. 1b). Analogously, determinants of the folding mechanisms are the rate of secondary structure formation (i.e., conformational change) and binding rate of intramolecular segments.

**Fig. 3:**
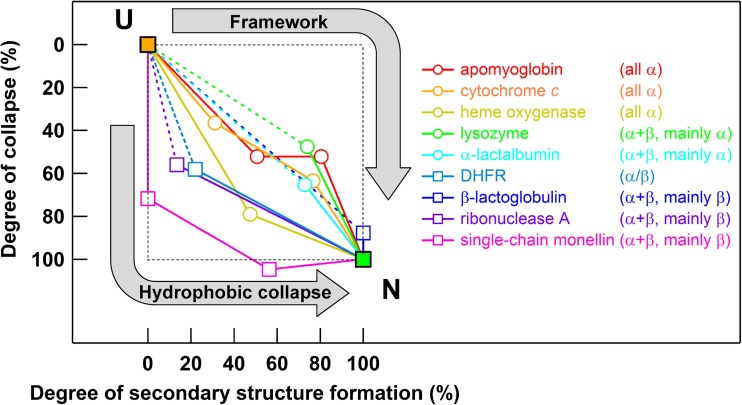
Folding trajectories of multi-subdomain proteins drawn in the simplified folding landscape. The horizontal and vertical axes show the degree of secondary structure formation, estimated from the change in circular dichroism intensity during folding, and degree of collapse, estimated from the change in the radius of gyration during folding, respectively. The unfolded state (*U*) and native state (*N*) are located at the *upper left* and *lower right* respectively. *Open circles and squares* show the location of folding intermediates of α-rich and β-rich proteins respectively. *Continuous and dotted lines* show the folding trajectories. Folding trajectories for the ideal conformational selection (*Framework*) mechanism and ideal induced-fit (*Hydrophobic collapse*) mechanism are indicated with *arrows*. Adapted with permission from Arai et al. (2007)

One method for discriminating which mechanism better explains protein folding reactions is to draw folding trajectories in the simplified folding landscape, in which one axis is the degree of secondary structure formation while the other axis is the degree of collapse (Arai et al. 2007) (Fig. 3). Here, the folding trajectory of the ideal framework model involves an expanded intermediate with a substantial amount of secondary structure, while that of the ideal hydrophobic collapse model involves a compact intermediate without any secondary structure (Fig. 1b). Experimentally characterized folding trajectories of multi-subdomain proteins showed that almost all folding pathways were in the lower left half of the landscape, indicating that multi-subdomain proteins fold by the hydrophobic collapse (induced-fit) mechanism rather than by the framework (conformational selection) mechanism (Arai et al. 2007) (Fig. 3). These results demonstrate that non-local hydrophobic interactions are more important than local secondary structure propensities early in the folding of multi-subdomain proteins. Furthermore, the results suggest that binding of intramolecular segments is faster than secondary structure formation, and competition between the rate of secondary structure formation and binding rate of intramolecular segments can determine the folding mechanisms. The rationale for these observations is that connections of interacting segments as a single chain increase the effective concentration between them and enhance their apparent binding rate. Experimental studies reported that protein hydrophobic collapse can occur within 60 ns (Sadqi et al. 2003), which is much faster than secondary structure formation (Kubelka et al. 2004; Muñoz and Cerminara 2016). If the second-order binding rate constant of intramolecular segments is 10^6^ M^−1^ s^−1^ and the effective concentration between them is 10 M (Robinson and Sauer 2000), hydrophobic collapse may occur on a time scale of 100 ns, which is consistent with experimental observations. Thus, although equilibrium and kinetic folding intermediates often have native-like secondary structures in a part of the molecule (Arai and Kuwajima 2000; Uversky and Fink 2002), non-specific hydrophobic collapse can occur before the formation of specific structures.

In addition to hydrophobic interactions, electrostatic interactions such as salt bridges can stabilize folding intermediates (Oliveberg and Fersht 1996). However, hydrophobic interactions are more important than electrostatic interactions in the folding of globular proteins. The presence of surface charge–charge interactions even decelerates a folding reaction, probably by restricting the ability to collapse (Kurnik et al. 2012).

The folding trajectories depicted in Fig. 3 also show that secondary structure contents in the native structure are related to the folding mechanisms. Proteins composed of mainly α-helices have folding trajectories involving concomitant compaction and secondary structure formation (Fig. 3), indicating rapid formation of α-helices. In contrast, proteins composed of mainly β-sheets display folding trajectories close to those of the ideal hydrophobic collapse model (Fig. 3), indicating slow formation of β-sheets. Therefore, secondary structure contents in native structure are closely related to the rate of secondary structure formation and thus determine the detailed folding mechanisms of multi-subdomain proteins (Arai et al. 2007).

In principle, protein folding reactions coupled with disulfide bond formation are essentially the same as those described above for disulfide-uncoupled folding. Experimental studies have shown that only native tertiary structures develop during oxidative folding if the refolding conditions are optimized (Wedemeyer et al. 2000), indicating that a native pair of cysteine residues comes in proximity resulting from hydrophobic collapse and native-like secondary structure formation during folding. Consistently, a disulfide-reduced protein can form an overall structure similar to the native state of the oxidized form (Redfield et al. 1999).

### Small single-domain proteins

Small globular proteins of less than 100 residues are typically composed of a single domain and fold in a two-state manner from the unfolded state to the native state without accumulation of an intermediate (Jackson 1998). The transition state between the unfolded and native state can be analyzed by Φ-value analysis (Fersht et al. 1992; Fersht and Sato 2004). Comprehensive Φ-value analysis has been performed for many small proteins, including chymotrypsin inhibitor 2 (CI2) (Itzhaki et al. 1995; Jackson 1998) and the B domain of protein A (Sato et al. 2004). The results showed that in the transition state, several non-local residues have high Φ-values and form specific interactions, while others have low Φ-values. The transition state had neither stable secondary structures nor compact structures (Plaxco et al. 1998), which is inconsistent with both the framework and hydrophobic collapse models. Because the number of hydrophobic residues is limited in small proteins, the binding rate between intramolecular segments is expected to be low. Additionally, the rate of secondary structure formation is expected to be low, as secondary structure elements have a small size and are less stable in small proteins. Thus, both hydrophobic collapse and secondary structure formation are less likely to occur early in the folding of small proteins. Rather, small proteins fold by a mechanism between the framework (conformational selection) and hydrophobic collapse (induced-fit) mechanisms, known as the *nucleation–condensation* mechanism (Fersht 1997). In this mechanism, a small number of non-local residues separated in an amino acid sequence form a specific hydrophobic interaction known as “a folding nucleus”. Formation of a folding nucleus is probably accompanied by formation of overall native-like secondary structure and backbone topology, although hydrogen bonds and tight packing of side chains have not yet formed. Once the critical interactions are formed, the remaining structure condenses rapidly around the nucleus to fold into a stable native structure.

Statistical analysis of the folding kinetics of small single-domain proteins showed that the logarithm of the folding rate, which corresponds to the free energy difference between the unfolded and transition state, Δ*G*_U-TS_, is negatively well-correlated with the contact order, a parameter that represents the native backbone topology of a protein (Jackson 1998; Plaxco et al. 1998, 2000; Ivankov et al. 2003; Kamagata et al. 2004). This correlation indicates that proteins in the all-α class, which have many local contacts and a lower contact order, fold faster, while those in the α/β and all-β classes, which have many non-local contacts and a higher contact order, fold more slowly. In other words, proteins that show a smaller (or larger) decrease in conformational entropy during folding have smaller (or larger) Δ*G*_U-TS_, suggesting that the free-energy barrier in the folding of small proteins corresponds to a decrease in the conformational entropy to form both a folding nucleus and overall native-like (but unstable) secondary structure and backbone topology in the transition state. Thus, the folding rate of small proteins is closely related to the rate of secondary structure formation.

Remarkably, a correlation between the folding rate and contact order has been observed, even for multi-subdomain proteins; both the folding rates from the unfolded state to the intermediate and from the intermediate to the native state were significantly correlated with native backbone topology, as represented by the absolute contact order (Kamagata et al. 2004; Kamagata and Kuwajima 2006). This suggests that the folding mechanisms of both small and multi-subdomain proteins are essentially identical. In fact, similar to the situation in multi-subdomain proteins, the rate of secondary structure formation and binding rate of intramolecular segments can determine the detailed folding mechanisms of small proteins. As described above, the folding rate of small proteins indicates the rate of secondary structure formation. In addition, the molecular size of the transition state indicates the binding rate of intramolecular segments. Statistical analysis showed that proteins in the all-α class have expanded molecular sizes in the transition state (Plaxco et al. 1998), indicating that α-helical proteins have faster rates of secondary structure formation but slower binding rates of intramolecular segments. This corresponds to the conformational selection (framework) mechanism. In contrast, proteins in the all-β class have compact dimensions in the transition state (Plaxco et al. 1998), indicating that β-sheet proteins have slower rates of secondary structure formation but faster binding rates of intramolecular segments. This corresponds to the induced-fit (hydrophobic collapse) mechanism. Thus, although small proteins generally fold by the nucleation–condensation mechanism, the detailed folding mechanism approaches the conformational selection (framework) mechanism when the rate of secondary structure formation is large and induced-fit (hydrophobic collapse) mechanism when it is small. These observations are consistent with those for multi-subdomain proteins (Arai et al. 2007). Thus, folding mechanisms of both small and multi-subdomain proteins can be integrated using the rate of secondary structure formation and binding rate of intramolecular segments.

The above considerations suggest that small proteins with many hydrophobic residues fold by the induced-fit (hydrophobic collapse) mechanism, as observed in the three-state folding of ubiquitin (Khorasanizadeh et al. 1996). Moreover, it is suggested that small proteins containing stable α-helices may fold by the conformational selection (framework) mechanism, as observed for the engrailed homeodomain that accumulates a folding intermediate with α-helices (Mayor et al. 2003).

By analogy, the nucleation–condensation mechanism, which is between the conformational selection and induced-fit mechanisms, has been observed in the coupled folding and binding of small IDPs. Whereas pKID has a low helical propensity particularly in the αB region, which makes a dominant contribution to the free-energy barrier of binding and binds KIX by induced-fit, c-Myb has a high helical propensity and binds KIX by conformational selection (Arai et al. 2015). This suggests that IDPs with helical propensities between those of pKID and c-Myb bind their partners through the nucleation–condensation mechanism. In addition, stabilization of helical structures by mutations may change the binding mechanism from nucleation–condensation to conformational selection. An interesting example is the synergistic folding of ACTR and the nuclear cofactor binding domain (NCBD) of CBP, which exist in the unfolded and molten globule states in isolation respectively, but fold into specific structures upon mutual binding (Dogan et al. 2013; Haberz et al. 2016). The Φ-values of the transition state in the coupled folding and binding of ACTR and NCBD are low, indicating that ACTR and NCBD synergistically fold via the nucleation–condensation mechanism. Indeed, helical propensities predicted by the AGADIR server (Muñoz and Serrano 1994) were 5.9% and 5.0% for ACTR and NCBD respectively, which are between those of the αB region of pKID (0.6%) and c-Myb TAD (40.6%). In addition, stabilization of ACTR by mutations resulted in a binding reaction by the conformational selection mechanism (Iešmantavičius et al. 2014), supporting the conclusion that wild-type ACTR binds NCBD by nucleation–condensation. Therefore, mechanisms of coupled folding and binding of IDPs that form helices upon binding may be predicted by their helical propensities.

### Subdomain-wise folding of large proteins

Multi-subdomain proteins are composed of several subdomains corresponding to small single-domain proteins. There are two ways to connect two small proteins as subdomains in a multi-subdomain protein. One is end-to-end tandem connection. Here, if two subdomains do not have mutual interactions and are stable enough to fold independently, both subdomains may fold by the nucleation–condensation mechanism (Arora et al. 2006). In contrast, if two subdomains interact with each other directly or indirectly through a rigid linker, both subdomains may affect the mutual folding reactions (Batey et al. 2008; Steward et al. 2012). The enzyme rhodanese, which has two similar subdomains tightly packed with each other, tends to misfold and requires molecular chaperones to correctly fold (Mendoza et al. 1991).

Another way of connecting two small proteins is to insert one subdomain (continuous insert) into a loop region of another subdomain (discontinuous parent). Domain insertion has been observed for 9% of domain combinations in the non-redundant structure database (Aroul-Selvam et al. 2004). If the insert subdomain is more stable than the parent subdomain, the insert may fold faster than the parent. In contrast, if the insert is unstable, the parent may fold faster than the insert. If both the insert and parent have similar amino acid sequences, folding reactions are more complicated; insertion of one CI2 into another CI2 resulted in destabilization of the double CI2, leading to the existence of two native conformers that folded and unfolded through two parallel pathways (Inaba et al. 2000).

For both ways of connecting two subdomains, subdomain-wise folding can occur, accumulating a folding intermediate in which one subdomain is partially folded and another is not. Such consideration is consistent with experimental results showing that one of the subdomains has a more organized structure than the others in the molten globule intermediate observed during the folding reaction of multi-subdomain proteins, including α-LA, lysozyme, apomyoglobin, barnase, and DHFR (Fersht 1993; Matagne and Dobson 1998; Arai and Kuwajima 2000; Arai et al. 2011; Dyson et al. 2017). However, because the number of hydrophobic residues is larger in multi-subdomain proteins than in small proteins, hydrophobic collapse rather than nucleation of a small number of hydrophobic residues tend to occur in multi-subdomain proteins, and thus compact molten globules with a localized native-like structure are observed.

**Fig. 4:**
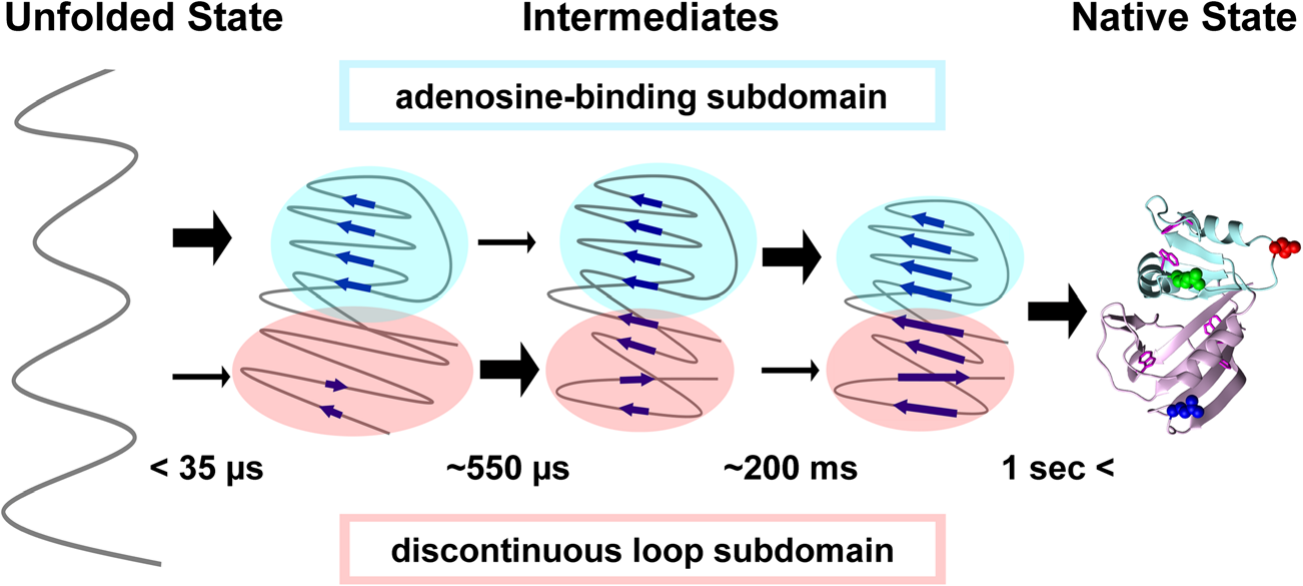
Subdomain-wise folding of DHFR. *Blue arrows* in the folding intermediates represent β-strands. *Thick and thin black arrows* respectively show that large and small conformational changes occur in the indicated subdomain during each phase. Adapted with permission from Arai et al. (2011)

Recent experimental studies of DHFR folding support the subdomain-wise folding mechanism (Arai et al. 2011) (Fig. 4). DHFR consists of two subdomains, a discontinuous loop subdomain (DLD) and continuous, adenosine-binding subdomain (ABD). Previous stopped-flow studies showed that the folding intermediate (I_5_) that formed within the dead time (~5 ms) of the measurement had a more ordered structure in the DLD than in the ABD. However, continuous-flow experiments combined with fluorescence resonance energy transfer measurement showed that the burst-phase intermediate (I_6_) that formed within the dead time (35 μs) of the measurement had a more compact structure in the ABD than in the DLD, and that compaction of the DLD to form the I_5_ intermediate occurred with a time constant of 550 μs (Arai et al. 2011). Thus, hierarchical assembly of DHFR was observed in which each subdomain independently folds, subsequently docks, and then anneals into the native conformation after an initial heterogeneous global collapse. This observation suggests that proteins with kinetic molten globule intermediates, in which discontinuous subdomains are more organized than continuous subdomains when observed by stopped-flow techniques, may fold through initial compaction of a continuous subdomain when observed by techniques with a shorter dead time. Progressive folding, beginning with a continuous subdomain and spreading to distal regions, shows that chain entropy is a significant organizing principle in the folding of multi-subdomain proteins and single-domain proteins (Arai et al. 2011).

Notably, recent studies of in vivo folding have shown that codon translation rates can profoundly impact the cotranslational folding process on the ribosome, indicating that protein folding is guided not only by the amino acid sequence but also by the RNA sequence (O’Brien et al. 2014). The presence of rare codon clusters at domain boundaries of proteins, which may efficiently enable domain-wise folding of large multi-domain proteins, is controversial (Deane et al. 2011).

### Theory of folding mechanisms of globular proteins

Theoretical studies of protein folding have proposed that the energy landscape of a protein has a funnel shape, and that an unfolded protein molecule folds into its native state by sliding down the surface of the energy landscape (Bryngelson et al. 1995; Dill and Chan 1997; Dinner et al. 2000; Shakhnovich 2006). Development of theoretical methods for predicting a folding energy landscape and native structure using only an amino acid sequence is one of the major goals of theoretical studies of protein folding (Dill et al. 2008). One of the most promising theoretical models describing protein folding mechanisms is the *Wako–Saitô–Muñoz–Eaton (WSME)* model (or island model) (Wako and Saitô 1978a, b; Muñoz and Eaton 1999; Sasai et al. 2016). The WSME model is a coarse-grained, statistical mechanical model of proteins and enables one to draw a free-energy landscape of a protein-folding reaction using information from the native structure. The model assumes that each residue adopts only the unfolded and native states and that two residues form a native-like contact only when the intervening residues between them are all in the native state. Thus, folding starts from local interactions between neighboring residues, and spreads to distal regions by the growth and coalescence of native-like segments. Moreover, this model considers only native contacts and thus reproduces folding reactions of hypothetical idealized proteins that can always fold into their native state (Gō 1983). Consequently, the WSME model guarantees that both the principle of minimal frustration and consistency principle that locally stable structure is consistent with the final folded, globally stable structure (Gō 1983; Bryngelson et al. 1995). Previous studies showed that the WSME model accurately explains the experimentally observed folding mechanism, i.e., the nucleation–condensation mechanism, of small single-domain proteins (Muñoz and Eaton 1999; Itoh and Sasai 2006; Sasai et al. 2016). These results suggest that real small proteins behave as ideal foldable proteins and that the consistency principle holds for small proteins.

Application of the WSME model to multi-subdomain proteins with end-to-end tandem connections has been successful in drawing free-energy landscapes of folding (Itoh and Sasai 2008, 2009). However, during the folding reactions of multi-subdomain proteins containing a subdomain insert, intermediates with localized native-like structures are frequently observed, in which a discontinuous parent subdomain is more organized than a continuous insert subdomain. Such folding behaviors cannot be described by the WSME model, as the model assumes that a discontinuous subdomain folds only after folding of the intervening subdomain. To solve this problem, a virtual linker between the N- and C-termini of DHFR, separated by 15 Å in the native state, was introduced in the *extended WSME (eWSME)* model (Inanami et al. 2014). Experimental studies in which both termini were connected by a linker showed that “circular” DHFR was more stable than wild-type “linear” DHFR, but that the folding behaviors under native conditions were unchanged by circularization (Arai et al. 2003b; Takahashi et al. 2007). Therefore, the eWSME model with a virtual linker may predict a free-energy landscape of folding that is applicable to a protein without a linker. Application of the eWSME model to DHFR predicted a folding pathway involving initial folding of the ABD and subsequent folding of the DLD, which is consistent with the experimental results (Inanami et al. 2014). Therefore, the eWSME model has been successfully used to explain the folding of a multi-subdomain protein containing a subdomain insert.

Although the original Ising-like Hamiltonian of the WSME model can explain the nucleation–condensation mechanism of folding for small single-domain proteins, the requirement of an additional Hamiltonian for linker introduction suggests that the original Hamiltonian is not sufficient to describe the cooperative hydrophobic collapse (induced-fit) mechanism of folding for multi-subdomain proteins, and that an additional term to enhance non-local (hydrophobic) interactions is necessary to formulate the consistency principle for multi-subdomain proteins. Future studies applying the eWSME model to many other proteins, optimizing linker introduction, and/or formulating variants of the WSME model may enable the prediction of free-energy landscapes of folding of all proteins. Moreover, because the forces responsible for folding and binding are common in globular proteins and IDPs, the WSME model is promising for developing a unified theoretical description of the mechanisms of folding and binding of all proteins. Indeed, the *allosteric WSME* model has been successfully applied to explain conformational selection and induced-fit mechanisms involved in allosteric transitions of proteins coupled with effector binding (Itoh and Sasai 2010, 2011; Sasai et al. 2016). Although future improvement in computer simulations may enable the reproduction of folding and binding reactions of all proteins, coarse-grained, statistical mechanical models are still required to determine the physics underlying these biological phenomena.

## Folding mechanisms of multimeric proteins

Elucidation of the folding mechanisms of multimeric proteins is also important, as most natural proteins exist as oligomeric complexes (Goodsell and Olson 2000). Multimer formation from fully unfolded monomers involves both folding and binding. Thus, overall folding mechanisms of multimeric proteins correspond to a combination of the folding mechanisms of monomeric globular proteins and coupled folding and binding mechanisms of IDPs. Many multimeric proteins fold by the induced-fit mechanism in which inter-subunit interactions induce subunit folding (Jaenicke 1987; Gloss and Matthews 1998; Jaenicke and Lilie 2000; Rumfeldt et al. 2008). This mechanism is observed when monomers are unstable and when segment-swapped dimers are formed (Rentzeperis et al. 1999; Topping and Gloss 2004). Large stabilization energies and highly cooperative folding transitions of multimeric proteins primarily result from inter-subunit interactions (Neet and Timm 1994; Arai et al. 2003a). There are also many examples of multimeric proteins that fold by the conformational selection mechanism, in which subunit assembly occurs after the formation of molten globule-like monomeric intermediates (Jaenicke 1987; Jaenicke and Lilie 2000; Svensson et al. 2006; Rumfeldt et al. 2008; Galvagnion et al. 2009; Noel et al. 2009). This mechanism tends to be observed when monomers are stable. Some multimers fold by an ideal conformational selection mechanism, in which formation of a binding-competent structure is a prerequisite for inter-subunit interactions (Topping and Gloss 2004). In such cases, monomeric intermediates are not kinetic traps, but rather productive, on-pathway intermediates. Both hydrophobic interactions and electrostatic interactions are important for the folding of multimeric proteins, as the reactions are accelerated by strengthening hydrophobic interactions and by removing electrostatic repulsion (Waldburger et al. 1996; Jelesarov et al. 1998; Dürr et al. 1999; Rentzeperis et al. 1999).

Reflecting the complexity of the native structures, larger multimers generally fold with more complicated folding mechanisms, involving both conformational selection and induced-fit mechanisms. Examples include sequential formation of monomeric and dimeric intermediates and the presence of parallel folding channels (Rumfeldt et al. 2008). Multimers larger than dimers may fold through formation of lower-order multimers and their assembly (Dürr and Bosshard 2000; Ali et al. 2003; Riechmann et al. 2005; Rumfeldt et al. 2008). Gp57A is a molecular chaperone for tail fiber formation of bacteriophage T4, and folds into a native hexameric structure by rapidly forming a trimeric coiled-coil intermediate (Matsui et al. 1997; Ali et al. 2003).

One of the best-studied multimers is the homodimeric coiled-coil peptide GCN4-p1. In the folding of GCN4-p1, partial helix formation precedes dimerization (Zitzewitz et al. 2000), indicating a conformational selection mechanism. Consistent with this, stabilization of helical structures accelerated the folding reaction (Zitzewitz et al. 2000). However, destabilization of the helices resulted in the induced-fit mechanism, in which the initial step is the binding of two unstructured monomers (Meisner and Sosnick 2004). These results support the notion that competition between the rate of conformational change and apparent rate of binding determines apparent reaction mechanisms. Interestingly, cross-linked variants of GCN4-p1 accumulate a folding intermediate formed by collision of two strands (Wang et al. 2005), supporting the view that connections of interacting segments increase the effective concentration between them and enable rapid association.

## Mechanisms of ligand binding

### Ligand-induced folding

Folding of globular proteins and IDPs can be coupled with binding to various ligands, including DNA/RNA (Spolar and Record 1994; Rentzeperis et al. 1999; Boehr et al. 2009; van der Vaart 2015), metals (Wittung-Stafshede 2002; Wilson et al. 2004; Li et al. 2015), osmolytes (Henkels et al. 2001), and cations/anions (Hagihara et al. 1993; Daniels et al. 2014, 2015). Many metalloproteins retain strong metal binding even in the unfolded state, indicating the induced-fit mechanism, but some metalloproteins must form well-defined metal-binding sites before metal binding, indicating the conformational selection mechanism (Wilson et al. 2004).

Ligand-induced folding is also observed for proteins used in folding studies. Binding of a heme to apomyoglobin induces folding of the F-helix (Eliezer and Wright 1996). *Escherichia coli* DHFR has four native structures (Jennings et al. 1993), one of which binds the cofactor NADPH by conformational selection (Dunn et al. 1978). α-LA and Ca^2+^-binding lysozymes form molten globule states in the absence of metals (Kuwajima 1989; Arai and Kuwajima 2000; Nakao et al. 2005). Because ligand binding is necessary for their folding, these proteins can be classified as IDPs. The Ca^2+^-binding site of α-LA is organized at the transition state of folding from the molten globule to the native state (Kuwajima 1989).

### Ligand binding to globular proteins

Studies of free proteins and protein–ligand complexes have demonstrated that proteins can fluctuate into conformations resembling those of the bound form, even in the absence of ligands, suggesting the conformational selection mechanism (Boehr et al. 2009). Ligand-binding reactions have been reported to occur by the conformational selection mechanism for many globular proteins, including DHFR, cyclophilin A, adenylate kinase, ribonuclease A, triose phosphate isomerase, ubiquitin, calmodulin, *lac* repressor, immunoglobulin, NCBD, maltose binding protein, and trypsin-like proteases (James and Tawfik 2003, 2005; Henzler-Wildman and Kern 2007; Tang et al. 2007; Lange et al. 2008; Loria et al. 2008; Boehr et al. 2009; Kjaergaard et al. 2010; Ma and Nussinov 2010; Nashine et al. 2010; Changeux 2012; Vogt et al. 2014). Adenylate kinase was previously thought to bind a ligand by the induced-fit mechanism, but was recently shown to occur by conformational selection using state-of-the-art methodologies to observe protein dynamics (Henzler-Wildman et al. 2007b). Even characteristic enzyme motions detected during catalysis were found to be already present in the free enzyme with frequencies corresponding to the catalytic turnover rates, suggesting that the protein motions necessary for catalysis are an intrinsic property of the enzyme (Eisenmesser et al. 2005; Boehr et al. 2006b). Furthermore, proteins from thermophilic bacteria have high optimal temperatures and function through flexible motions at high temperatures; however, catalytic activity is reduced at low temperatures by the repression of fluctuations (Závodszky et al. 1998; Wolf-Watz et al. 2004). These results support the importance of fluctuations and dynamics in protein function.

The dominance of the conformational selection mechanism indicates that the apparent ligand-binding rate of the weakly binding conformation is lower than the fast rate of conformational change, occurring on microsecond to millisecond time scales. Specific complementarity in shape and polarity between a protein and ligand is typically necessary for tight binding, and slight changes in protein conformations by fluctuations cause steric hindrance of binding.

The enzyme reaction cycle of *E. coli* DHFR goes through five intermediate states (Fierke et al. 1987; Schnell et al. 2004; Boehr et al. 2006a, b; Hammes-Schiffer and Benkovic 2006). NMR relaxation dispersion experiments showed that all five intermediate states can access conformations resembling those of the next step in the reaction cycle, indicating that all steps in the reaction cycle occur by the conformational selection mechanism (Boehr et al. 2006b). Surprisingly, the next state in the reaction cycle is already prepared as an excited state, and this efficient reaction cycle is encoded in the amino acid sequence of only 159 residues. Human DHFR has more flexible structures and higher activity than *E. coli* DHFR (Bhabha et al. 2013), supporting the link between protein dynamics and enzymatic catalysis. Thus, the intrinsically flexible nature of DHFR is essential for the enzymatic reaction, which is manifested in multiple native structures in the apo form of *E. coli* DHFR (Jennings et al. 1993).

Although conformational selection is the prevailing mechanism, the presence of high concentrations of a ligand can shift the binding mechanism toward the apparent induced-fit mechanism (Hammes et al. 2009; Greives and Zhou 2014). Moreover, the ideal induced-fit mechanism is observed for the ligand binding of HIV-1 protease, in which the conformational change from the semi-open to the closed form occurs after ligand binding, as the ligand-binding site is sequestered in the closed form and access of a ligand to the buried binding site is sterically prohibited (Hornak and Simmerling 2007). Recently, an increasing number of reports showed that proteins bind ligands by the sequential conformational-selection and induced-fit mechanism, in which primary binding by conformational selection is followed by conformational optimization by induced-fit (James and Tawfik 2003, 2005; Tang et al. 2007; Wlodarski and Zagrovic 2009; Wang et al. 2013a).

The physical origin of catalytically important collective motions in slow time scales (microsecond to millisecond) is the local hinge motions in fast time scales (picosecond to nanosecond) (Henzler-Wildman et al. 2007a), and is related to the average time required to sample the configurations that are conductive to chemical reactions, which occur on femtosecond to picosecond time scales (Ma and Nussinov 2010; Hammes et al. 2011).

### Stability–activity trade-off

The conformational selection mechanism assumes that equilibrium exists between the binding-incompetent and binding-competent conformations, while the induced-fit mechanism assumes that conformational change occurs from the weakly binding conformation to the tightly binding conformation. In both cases, the conformation with lower affinity for a ligand often corresponds to the native structure of the free protein. Thus, introduction of mutations that stabilize the native state of a free protein decelerates the transition to the conformation with higher affinity for a ligand, leading to a decrease in the ligand-binding rate and, for an enzyme, a decrease in catalytic activity. This observation is known as the “stability–activity trade-off” (Siddiqui 2017). From a structural perspective, the ligand-binding sites of proteins often contain conformational strain, hydrophobic surface exposure, and/or electrostatic repulsion caused by residues with the same charges. Thus, interactions in the native state of a protein are not optimized but can confer flexibility and fluctuation to lower-affinity conformations. Consequently, mutations at ligand-binding sites can optimize the stability of a native protein by removing unfavorable interactions, but concomitantly reduce the affinity for a ligand, leading to a stability–activity trade-off (Shoichet et al. 1995; Wang et al. 2002; Thomas et al. 2010; Yokota et al. 2010; Klesmith et al. 2017). In contrast, mutations that stabilize the binding-competent conformation and/or destabilize the binding-incompetent conformation—that is, the native structure of a free protein—should increase affinity and activity. IDPs are extreme cases of protein destabilization for tight binding.

The stability–activity trade-off can be viewed as competition between intramolecular and intermolecular interactions. By analogy, this corresponds to the interplay of local and non-local interactions in the folding of monomeric proteins. Previous studies reported that whereas favorable native-like local interactions can accelerate folding reactions (Viguera et al. 1997), further stabilization of secondary structure decelerates the folding rates (Chiti et al. 1999). Moreover, if non-native local structures are stable, they can be observed in kinetic folding intermediates, but not in the final native state (Forge et al. 2000). Thus, both the balance between local and non-local interactions and minimization of non-native local interactions are important for optimizing protein stability and folding speed (Muñoz and Serrano 1996). This suggests that the balance between intramolecular and intermolecular interactions is important for protein stability and activity.

## Unified mechanisms of protein folding and binding

We have described the mechanisms of coupled folding and binding of IDPs, folding of small and multi-subdomain proteins, folding of multimeric proteins, and ligand binding of globular proteins. All mechanisms are well-explained using the conformational selection and induced-fit mechanisms as well as the nucleation–condensation mechanism, which is intermediate between them, suggesting that we can integrate the understanding of folding and binding mechanisms of globular proteins and IDPs.

In general, reaction mechanisms are determined by competition of the fluxes of the conformational selection and induced-fit pathways. Accumulating evidence has shown that both the rate of conformational change and apparent rate of binding between interacting elements can determine reaction mechanisms. The former is affected by both the forward rate (P_weak_ to P_tight_) and reverse rate (P_tight_ to P_weak_), while the latter is affected by the second-order binding rate constant, first-order dissociation rate constant, and protein and ligand concentrations (see Fig. 1a). If the conformational change from P_weak_ to P_tight_ is faster than the ligand binding of P_weak_, the reaction tends to occur through the apparent conformational selection mechanism. In contrast, if the ligand binding of P_weak_ is faster than the conformational change, the reaction tends to occur through the apparent induced-fit mechanism. Because the folding of monomeric proteins can be regarded as the binding of intramolecular segments accompanied by secondary structure formation, the rate of conformational change corresponds to the rate of secondary structure formation and the binding rate corresponds to the rate of binding between intramolecular segments. Instead of protein and ligand concentrations, the effective concentration between intramolecular segments affects the apparent binding rate in protein folding.

Because most IDPs contain unstable secondary structure elements, the secondary structure formation of free IDPs is slower than binding with partners. Therefore, coupled folding and binding reactions of IDPs are dominated by the induced-fit mechanism. In contrast, ligand-binding reactions of globular proteins are dominated by the conformational selection mechanism, as the conformational change is fast and because the weakly binding conformation of a protein has low affinity for a ligand because of the lack of specific complementarity between them. For both IDPs and globular proteins, if the rate of conformational change and rate of binding are comparable, the nucleation–condensation mechanism is observed.

In the case of folding reactions of monomeric proteins, the rate of secondary structure formation and binding rate of intramolecular segments can determine the folding mechanisms. Multi-subdomain proteins fold by the induced-fit (hydrophobic collapse) mechanism because the connection of interacting segments increases the effective concentration between them and accelerates their mutual binding. Indeed, protein hydrophobic collapse can occur much faster than secondary structure formation. In contrast, small proteins typically fold by the nucleation–condensation mechanism. Secondary structure elements with short length and small numbers of hydrophobic residues in small proteins preclude the conformational selection (framework) mechanism and induced-fit (hydrophobic collapse) mechanism respectively. However, for both small and multi-subdomain proteins, the folding mechanism approaches the conformational selection (framework) mechanism or induced-fit (hydrophobic collapse) mechanism if secondary structure formation is fast or slow respectively. Generally, α-helical proteins fold faster than β-sheet proteins. Thus, the secondary structure contents in native structures can determine the detailed folding mechanisms. Therefore, the folding mechanisms of both small and multi-subdomain proteins are essentially identical, except that lower or higher numbers of hydrophobic residues lead to nucleation or collapse of hydrophobic residues in small or multi-subdomain proteins respectively.

Both long-range electrostatic interactions and non-local hydrophobic interactions are essential for determining the binding rate, while local secondary structure propensities are important for determining the rate of conformational change. For intermolecular interactions, electrostatic attractions are more effective than hydrophobic interactions in diminishing the distance between interacting elements. In protein folding, non-local hydrophobic interactions are more important than local secondary structure propensities early in the folding reaction.

Folding mechanisms of multimeric proteins are consistent with those of monomeric globular proteins and IDPs. Depending on the stability of subunit structures, many multimeric proteins fold by both the conformational selection mechanism, in which subunit assembly occurs after formation of molten globule-like monomeric intermediates, and induced-fit mechanism, in which inter-subunit interactions induce subunit folding.

**Fig. 5:**
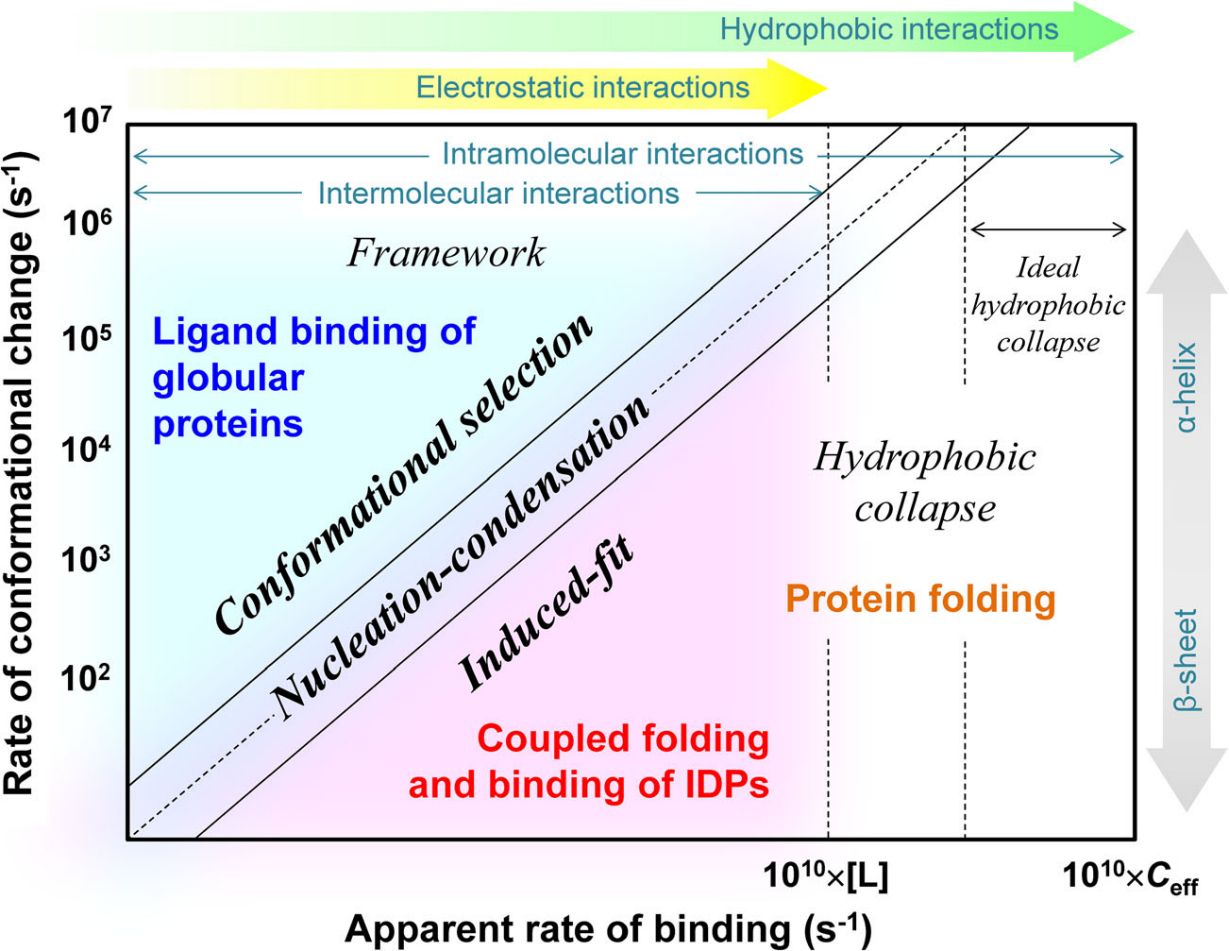
Apparent dependence of the folding and binding mechanisms of globular proteins and IDPs on both the rate of conformational change and apparent binding rate of interacting elements. *[L]* and *C*_eff_ denote the free ligand concentration and effective concentration for intramolecular interactions respectively. See text for details

Figure 5 summarizes the apparent dependence of the folding and binding mechanisms of globular proteins and IDPs on both the rate of conformational change (vertical axis) and apparent binding rate of interacting segments (horizontal axis). The rate limit of conformational change is the fastest folding rate of stable α-helices and β-hairpins (~10^7^ s^−1^). The upper limit of the apparent binding rate is estimated as ~10^10^ M^−1^ s^−1^ multiplied by a free ligand concentration [L] for intermolecular interactions and ~10^10^ M^−1^ s^−1^ multiplied by an effective concentration *C*_eff_ for intramolecular interactions. The reaction mechanism will be the induced-fit if the apparent binding is fast and conformational change is slow. In contrast, the reaction mechanism will be conformational selection if the apparent binding is slow and conformational change is fast. If both rates are comparable, the reaction mechanism will be the nucleation–condensation mechanism. Whereas coupled folding and binding reactions of IDPs are located on the lower right side in Fig. 5, ligand-binding reactions of globular proteins are located on the upper left side. Folding reactions of monomeric and multimeric proteins can be located over a wide region, but are mainly located on the lower right side. Fast binding (compaction) may require large numbers of hydrophobic residues, resulting in folding mainly by the induced-fit (hydrophobic collapse) mechanism. When the intramolecular binding rate exceeds the rate limit of conformational change, the folding reaction will occur by the ideal induced-fit (hydrophobic collapse) mechanism.

In summary, the folding and binding mechanisms of globular proteins and IDPs obey the same general principle and can be integrated into a unified understanding. Elucidation of the mechanisms of folding and function of proteins and development of theoretical models to explain these mechanisms will profoundly impact the rational design of proteins for medicine and industry. The coarse-grained, statistical mechanical models explaining the mechanisms of folding reactions and allosteric transitions are promising for a unified theoretical description of folding and binding mechanisms of globular proteins and IDPs. Although many studies are needed to clarify these mechanisms, comparison of the mechanisms between various reactions will improve the understanding of each mechanism, and may enable a unified understanding to be developed that is applicable to all proteins.
